# Evaluation of 3-dimensional left ventricular velocities with cardiac MR imaging using navigator gated high temporal resolution tissue phase mapping

**DOI:** 10.1186/1532-429X-11-S1-P278

**Published:** 2009-01-28

**Authors:** Ion Codreanu, Cameron J Holloway, Oliver J Rider, Steffen E Petersen, Matthew D Robson, Bernd A Jung, Stephen J Golding, Stefan Neubauer, Kieran Clarke

**Affiliations:** 1 University of Oxford Centre for Clinical Magnetic Resonance Research, Headington, Oxford, UK; 2 Department of Diagnostic Radiology, Medical Physics, University Hospital, Freiburg, Germany; 3 MRI Centre, Oxford Radcliffe Hospital, University of Oxford, Headington, Oxford, UK; 4 Department of Physiology, Anatomy and Genetics, University of Oxford, Oxford, UK

**Keywords:** Myocardial Velocity, Cardiac Magnetic Resonance Examination, Tissue Phase Mapping, Arterial Pressure Wave, Left Ventricular Base

## Introduction

Identification of regional myocardial dysfunction with adequate temporal and spatial resolution is important for diagnosis and management of cardiovascular disease. Assessment of regional myocardial velocities allows 3-dimensional quantification of left ventricular function and carries independent prognostic information.

## Purpose

The aim of this study was to use navigator gated tissue phase mapping with high temporal resolution to obtain accurate normative data of left ventricular velocities, to assess normal patterns of left ventricular motion and to evaluate the reproducibility of this technique.

## Methods

Cardiac magnetic resonance examinations were performed on a 1.5 Tesla MR clinical scanner (Sonata; Siemens Medical Solutions, Erlangen, Germany). Cardiac gated, phase contrast measurements using respiratory navigator with a temporal resolution of 13.8 ms were obtained on two separate occasions on 13 healthy male subjects (age 22 ± 3 years) three weeks apart. Three short axis images were obtained for the left ventricular base, mid-ventricle and apex. Each short axis acquisition took approximately 3 – 5 minutes, with an average of 60 – 70 phases per cardiac cycle. Subsequent normative velocities were obtained for radial, circumferential and longitudinal motion.

## Results

**Figure 1:**
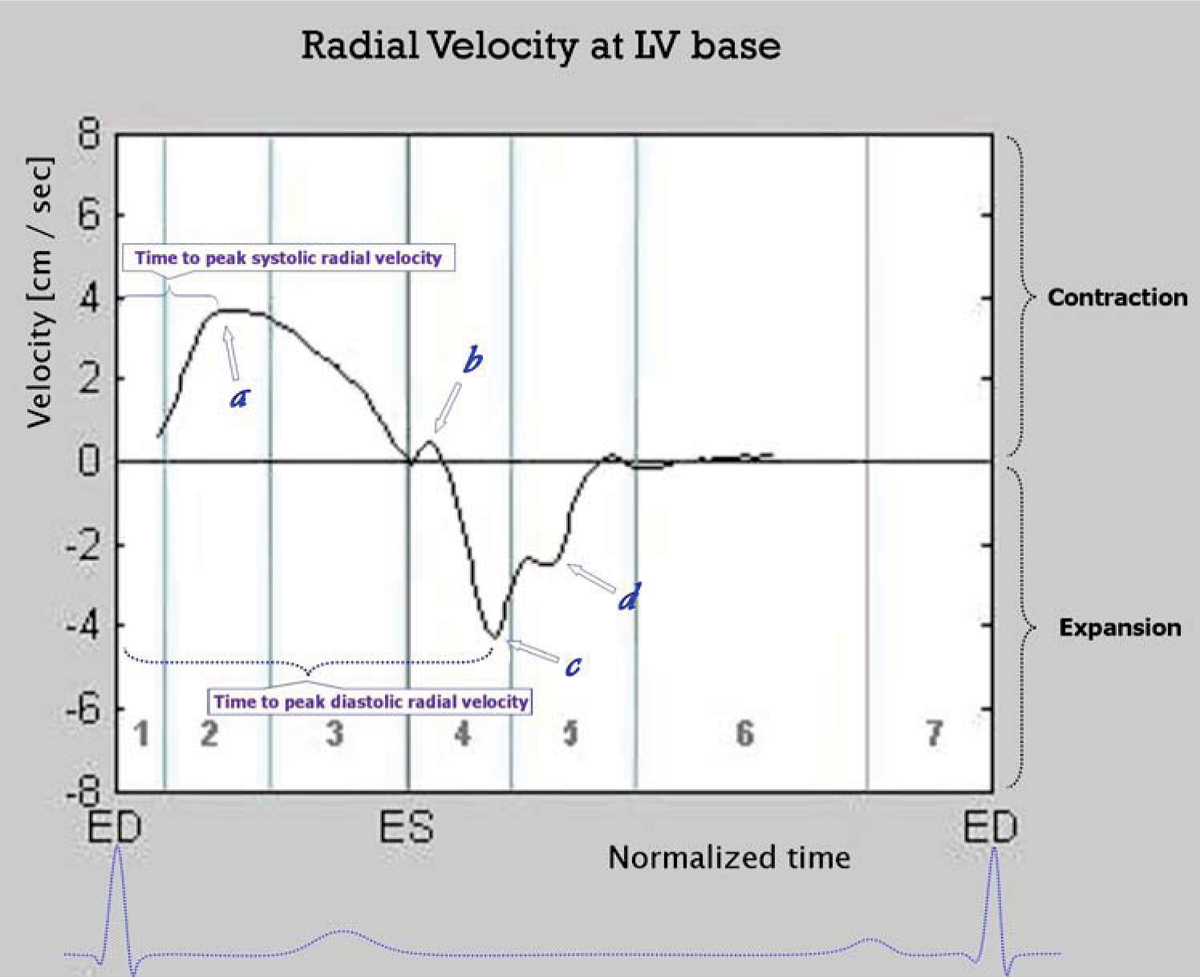
**Radial velocity graph at left ventricular base**. 1 – isovolumetric contraction, 2 – rapid ejection, 3 – reduced ejection, 4 – isovolumetric relaxation, 5 – rapid filling, 6 – diastasis, 7 – atrial contraction, ED – end diastole, ES – end systole, a – peak systolic radial velocity, b – upward directed notch in early diastole, c – peak diastolic radial velocity, d – additional thrust of outward radial movement during rapid ventricular filling.

High temporal resolution tissue phase mapping analysis allowed visualization of additional details of ventricular motion that have not been observed with breath-held lower temporal resolution tissue phase mapping [1]. Changes in the direction of radial, circumferential and longitudinal movement were apparent after repolarization. An additional wave of outward radial movement, corresponding to the timing of the third heart sound, was observed at the left ventricular base during rapid ventricular filling (Figure 1d). An upward directed notch in early diastole (Figure 1b) similar to that on arterial pressure wave was recorded on radial and longitudinal velocity graphs. The longitudinal velocity graph showed a downward movement of the whole ventricle during rapid ejection, followed by an upward displacement after repolarization. The commencement of left ventricular untwisting was also noticed after repolarization. A normative database for radial, circumferential and longitudinal velocities for epicardial, myocardial and endocardial layers as well as for peak times was obtained using this technique. Repeated measurements showed good agreement on Bland-Altman plots.

## Conclusion

Navigator gated tissue phase mapping with high temporal resolution is a reproducible technique to evaluate detailed regional myocardial wall motion and provides valuable and comprehensive information for quantifying myocardial velocities. The technique allowed us to obtain new details of left ventricular motion patterns and may prove to be a powerful tool for assessing subtle changes in myocardial function in cardiac disease.
